# Hypochloraemia and Worse Clinical Outcome in Critically Ill Patients

**DOI:** 10.1100/2012/234628

**Published:** 2012-09-25

**Authors:** Rainer Gatz, Paul Elbers

**Affiliations:** ^1^ICU, Herlev Hospital, 2730 Herlev, Denmark; ^2^ICU, Onze Lieve Vrouwe Gasthuis, Amsterdam, The Netherlands

Tani et al. recently found an association between hypochloraemia and worse clinical outcome in critically ill patients, albeit not as an independent risk factor [1].

 The authors were unable to find any explanation for this association. From clinical experience we could invoke three possible causes: use of NaHCO_3_, use of loop diuretics, or renal compensation for acidosis, all of which might be more prevalent in sicker patients. One of the tables presents data about the “strong ion gap” (SIG) in the three subgroups of hyper-, normo-, and hypochloraemic patients, with mean values of, respectively, 3.5 versus 4.8 versus 6.2 mEq/L. The corresponding *P*-value is <0.0001. The authors do not mention this in their discussion section, though. While the value of SIG as an independent risk factor for poorer clinical outcome may not be proven, there are a few studies that indicate such a relation [2–6]. The link between hypochloraemia and worse clinical outcome might thus be that hypochloraemia is an indicator of higher SIG values. It would be highly interesting if the authors could contribute to this aspect of their data.
